# A qualitative investigation into knowledge, beliefs, and practices surrounding mastitis in sub-Saharan Africa: what implications for vertical transmission of HIV?

**DOI:** 10.1186/1471-2458-7-22

**Published:** 2007-02-23

**Authors:** Manuela De Allegri, Malabika Sarker, Jennifer Hofmann, Mamadou Sanon, Thomas Böhler

**Affiliations:** 1 Department of Tropical Hygiene and Public Health, University of Heidelberg, Germany; 2 Institute of Ethnology, University of Heidelberg, Germany; 3 Centre de Recherche en Santé de Nouna, Nouna, Burkina Faso; 4 Department of Virology, University of Heidelberg, Germany

## Abstract

**Background:**

Mastitis constitutes an important risk factor in HIV vertical transmission. Very little, however, is known on how women in sub-Saharan Africa conceptualise health problems related to breastfeeding, such as mastitis, and how they act when sick. We aimed at filling this gap in knowledge, by documenting the indigenous nosography of mastitis, health seeking behaviour, and remedies for prophylaxis and treatment in rural sub-Saharan Africa.

**Methods:**

The study was conducted in the Nouna Health District, rural Burkina Faso. We employed a combination of in-depth individual interviews and focus group discussions reaching both women and *guérisseuers*. All material was transcribed, translated, and analysed inductively, applying data and analyst triangulation.

**Results:**

Respondents perceived breast problems related to lactation to be highly prevalent and described a sequence of symptoms which resembles the biomedical understanding of pathologies related to breastfeeding, ranging from breast engorgement (stasis) to inflammation (mastitis) and infection (breast abscess). The aetiology of disease, however, differed from biomedical notions as both women and *guerisseurs *distinguished between "natural" and "unnatural" causes of health problems related to breastfeeding. To prevent and treat such pathologies, women used a combination of traditional and biomedical therapies, depending on the perceived cause of illness. In general, however, a marked preference for traditional systems of care was observed.

**Conclusion:**

Health problems related to breastfeeding are perceived to be very common in rural Burkina Faso. Further epidemiological research to assess the actual prevalence of such pathologies is urgently needed to inform the design of adequate control measures, especially given the impact of mastitis on HIV vertical transmission. Our investigation into local illness concepts and health care seeking behaviour is useful to ensure that such measures be culturally sensitive. Further research into the efficacy of local customs and traditional healing methods and their effect on viral load in breast milk is also urgently needed.

## Background

In sub-Saharan Africa (SSA), where breastfeeding constitutes the most common infant feeding practice [1-5], postnatal transmission of human immunodeficiency virus (HIV) through breastfeeding represents at least 24% (and possibly as much as 42%) of overall mother-to-child transmission (MTCT) of HIV [6]. The infants' risk of getting infected through breastfeeding appears to be highest in the first weeks of life and to be strongly associated with HIV viral load in breast milk [7]. In turn, recent studies have demonstrated that the presence of mastitis, an inflammatory process in the breast, is associated with an increase in HIV viral load in breast milk [8-12]. Therefore, mastitis is considered to be an important independent risk factor increasing HIV vertical transmission [13].

The literature suggests that mastitis represents just one of several pathologic states from which breastfeeding mothers may suffer. Medical problems linked to breastfeeding comprise a continuous spectrum of pathologic states ranging from breast engorgement due to reduced milk flow (stasis) to clinical mastitis (an inflammatory process in the breast producing localized tenderness, redness, and heat, together with systematic reactions of fever, malaise) [14], which may extend to breast abscess [13]. Sub-clinical mastitis has been identified by several researchers as a stage of the disease in which women do not complain about any subjective signs or symptoms except reduced milk flow but have a particular biochemical composition of breast milk [13]. In such cases, laboratory analysis of expressed breast milk revealed an elevated breast milk leukocyte count, an increase in the concentration of pro-inflammatory cytokines, and an increase in sodium or the sodium-potassium-ratio [13,15-17].

The literature further suggests that the occurrence of mastitis and/or breast inflammations in general is common both in resource-rich and in resource-poor settings. Clinical mastitis was diagnosed in 10% of American women during the first three months of lactation [18] and in 17% of Australian women during the first six months of lactation [19]. In a recent survey in Turkey, 80 (71%) out of 112 lactating women reported having suffered from breast problems (engorged breast, tenderness, and pain) in the two months postpartum [20].

Accurate information on the overall prevalence of breast inflammations and in particular of mastitis in SSA is not available. The most reliable estimates are derived from measurements among HIV-infected women enrolled in prevention of mother-to-child transmission (PMTCT) programs. In Kenya, 11% of HIV-infected women were diagnosed with mastitis and 12% with breast abscess [11]. A study in Malawi indicated that approximately 27% of HIV-infected women had experienced at least one episode of sub-clinical mastitis, defined as elevated breast milk leukocyte count, in the first year postpartum [16]. In Tanzania, sub-clinical mastitis, defined as elevated milk sodium-to-potassium-ratio, was diagnosed among 13% and 11% of women respectively one month and three months postpartum [15].

Even if the natural history and the clinical importance of sub-clinical mastitis remain unclear [15], these findings yield important implications for the design of PMTCT programs. In SSA, exclusive breastfeeding (EBF) followed by early weaning has been identified as the preferred infant feeding practice [21]. Given the poor hygienic conditions and the price of milk substitutes in fact, EBF followed by early weaning is generally considered to bear greater benefits than costs [22-27]. Thus, given the increased risk of HIV transmission associated with the presence of breast inflammations and mastitis, prophylaxis, early diagnosis, and treatment of such conditions have been proposed as an additional PMTCT strategy in resource-limited settings [28].

In spite of the role played by mastitis in the transmission of HIV and in spite of the fact that the first studies conducted in SSA have indicated the prevalence to be quite high among HIV-infected women [11,16], public health scientists have channelled little efforts towards understanding how women frame health problems related to breastfeeding and how they deal with them. With the exception of one ethnographic study by Alfieri and Taverne [29], little is known on local illness concepts and health care seeking behaviour in relation to breastfeeding problems in SSA. This study, conducted exclusively on two ethnic groups in West Africa, the Mossi and the Bobo Madare, revealed that women recognise as relevant to their everyday life health problems associated with breastfeeding. Women differentiate relatively simple problems linked solely to reduced milk production from more severe problems described as real breast pathologies. The study also revealed that women distinguish between natural and unnatural causes of disease and that they alternatively use traditional and modern medical remedies.

A comprehensive understanding of local illness concepts and health care seeking behaviour relevant to public health practice is still clearly missing, although essential to design effective, yet culturally sensitive PMTCT programs. This paper draws from the results of a qualitative study which preceded and informed the introduction of a PMTCT program in rural Burkina Faso. We aimed at: (a) documenting local illness concepts and the indigenous nosography of breast pathologies during lactation; (b) documenting women's health seeking behaviour; and (c) describing available remedies for prophylaxis and treatment.

## Methods

The field work was conducted in the Nouna Health District (located approximately 300 km from the capital Ouagadougou) between December 2002 and March 2003 within the framework of a larger anthropological study exploring local beliefs and practices related to breastfeeding. The methodology has been described in detail elsewhere [30].

In brief, we collected data using both focus group discussions (FGD) and in-depth individual interviews with women who were purposively selected on the basis of their experience with breastfeeding [31]. Given our focus on mastitis and HIV, we included women who reported having experienced lactating problems at least once in their lifetime. Women to be interviewed were identified at informal women's gatherings by JH with the assistance of a key informant. In addition, we conducted in-depth individual interviews with local *guérisseurs*, i.e. indigenous health practitioners ranging from herbalists to diviner mediums. We continued data collection until we reached saturation and redundancy [32].

JH conducted all interviews personally with the assistance of two translators, one working in Djoula and one in Bwamu. Before proceeding with the interview, JH explained the purpose and relevance of the study and sought the women's explicit verbal consent. Information was solicited through a series of semi-structured open-ended questions. JH and TB developed the interview guides for both the individual interviews and the FGD. The interview guides touched on different aspects of the culture and practice of breastfeeding. With specific reference to the themes addressed in this publication, the interview guide explored how women and *guérisseurs *construct and define what constitutes a lactating problem and what prevention and treatment options they resort to in case of need.

All material was recorded, transcribed, and translated into French by trained translators. We carried out the analysis on the French text, translating into English only the material which appears in our publications. We analysed the data inductively. We applied analyst triangulation as two independent researchers, JH and MDA, read the material separately and only compared and converged findings at a later stage [31]. Afterwards, we discussed the interpretation and the policy relevance of the findings among all authors. The systematic comparison of findings across data sources, women and *guérisseurs*, and between the individual interviews and the FGD provided an additional source of triangulation [31].

The study was approved by the Ethics committee of the Faculty of Medicine of the University of Heidelberg, Heidelberg, Germany, and by the Nouna Ethics Committee, Nouna, Burkina Faso.

## Results

We interviewed 38 women, as the result of 32 individual interviews and 2 FGD, and 5 *guérisseurs*. In addition, JH was invited to attend a meeting held among *guérisseurs *and had the opportunity to pose additional questions on such occasion. The respondents, whose age ranged from 17 to 80, were representative of all local major ethnic groups in the area: Marka, Bwaba, Mossi, Peuhl, and Samo. The vast majority of women were uneducated, were married, and as a source of income, they engaged in small-scale commercial activities.

The presentation of the findings is organized in three sections. For each reported verbatim quotation, we indicate the respondent's age and ethnicity. We explicitly indicate when quotations report the *guérisseurs' *speech. In addition, we have included a case study, Salima's story [see additional file 1] with the aim of allowing the reader to gain a better understanding of how a typical woman in Nouna perceives and defines her lactating problem and how she is likely reach a decision regarding health care seeking. We wish to point out that Salima is a fictional name used to protect the identity of the woman originally reporting the story. Given that the woman was illiterate, consent to use the information she shared with us was obtained verbally.

### Local illness concepts and indigenous nosography

Women perceived breast problems related to lactation to be highly prevalent. They indicated that every second breastfeeding mother experiences some sort of problem. They reported that the most frequently encountered problem is inappropriate lactation, defined as the production of insufficient quantities of milk. Women explained that such problems are addressed within one's community by modifying dietary habits or by resorting to the use of herbal infusions.

The emergence of one of several additional physical symptoms marked the differentiation between a resolvable lactation difficulty and an actual health problem which requires professional attention. Depending on the language they were most familiar with, respondents used a variety of words to define health problems of the breast related to lactation, "*siindimibanaw*" (Djoula), *"biis-guija" *(Mooré), and "*dindin*" (Bwamu), and clearly recognized their potential to constitute a threat to a woman's well-being. Both women and *guérisseurs *consistently described the same set of symptoms and the same sequence: (a) itching of the breast; (b) either a slow milk flow or a complete absence of milk flow or continuous dripping; (c) swelling of the mammary glands accompanied by an inflammation and at times by fever; (d) a painful breast abscess.

" I had an inflammation, then some milk dripping, then swelling, then an abscess, and in the end, a wound" (22, Marka)

"... the illness can persist leading up to death" (20, Samo)

"There are different forms of diseases of the breast among breastfeeding women. First, there can be little absence of milk ... then a swelling of the breast... then, there can be an inflammation, which can even turn into an abscess" (Guérisseur 2, Mossi)

Both women and *guérisseurs *identified two sources of illness: (a) breast problems due to "natural causes" with an observed cause-effect relationship, and (b) breast problems resulting from the action either of another human being, a sorcerer or a *marabou*, or of a nonhuman "force", such as a deity.

"The illness might be natural...or might be caused by sorcery" (22, Marka)

The respondents indicated that "natural" breast problems can arise as the result of inadequate breastfeeding practices or of a parasitic contamination. Adequate breastfeeding entailed both ensuring that the child correctly sucks the whole nipple and respecting traditional norms and behaviours related to motherhood.

"If the child sucks the breast from the side (*inadequately*), this can cause the illness" (24, Marka)

"It seems that there are parasites that can cause these problems" (19, Marka)

In addition, both women and *guérisseurs *mentioned mental and physical distress as well as no respect of basic hygienic conditions as additional "natural" causes of breast problems. They recognised that awareness and respect of hygienic conditions represent a recent development.

"Before women took no hygienic precautions ... Today, women take all precautions. When you return from town ... you must wash your hands and your breast before breastfeeding" (30, Mossi)

Breast problems resulting from the action of another human being were explained in relation to unsettled jealousy or envy between people.

" In my case, (*the breast problem*) is a spell thrown by my old boyfriend. When I refused to marry him, he told me that I would have never had children and I would have died young. This has not happened, but with each delivery (*each child*), I have breast problems" (22, Marka)

"A sorcerer can induce any breast problem in a woman to hurt her" (Guérisseur 3, Bambara)

### Health care seeking behaviour

To treat their breast problem, 17 women had consulted a *guérisseur*, 8 had gone to the hospital, 2 had used home treatment, and 11 had consulted several practitioners at different moments through their illness. Most women had first attempted to solve the problem at home, resorting to their family tradition of pharmacopoeia. In addition, women had adapted their breastfeeding behaviour preferring the sick breast above the healthy one as long as lactation was possible.

The choice of provider, traditional or modern, depended on the woman's socio-demographic profile, her economic status, and on her perception of the cause and the severity of the illness. Ethnicity played a role, with strict Muslim Mossi women preferring the *marabou *(the Muslim healer) above any other *guérisseur*. Younger women generally preferred modern above traditional medicine. Women consistently reported that breast problems which result from sorcery can only be treated by *guérisseurs*, while "natural" breast problems can be treated by both *guérisseurs *and modern health practitioners, leaving the choice to the woman's individual preference. They recognised, however, that the application of user fees often induces women to resort to traditional medicine even in instances when they would in fact prefer modern medicine. Women frequently reported seeking care at the hospital only once in need of a surgical intervention to remove the abscess.

"When it is a spell that someone threw on you, traditional medicine is more effective. When it is a natural disease, modern medicine is more effective" (22, Marka)

"Traditional medicine helped me a lot. Modern medicine only took care of the abscess" (30, Samo)

"I did not have the money to go to the hospital, so I did the incision at home and applied some traditional remedy" (35, Mossi)

In addition, women's choice of provider appeared to be heavily conditioned by the opinion and the will of other family members, in particular the parents and the husband.

"My parents refused that I consult the hospital, because they trust that traditional medicine is effective" (33, Marka)

"... because it is him (*my husband*) who cares for me, I can't decide to consult a *guérisseur *without his consent" (24, Bwaba)

### Traditional prophylaxis and treatment remedies

The *guérisseurs *shared women's opinion that the cause of the breast problem determines the treatment to be applied. In particular, the *guérisseurs *insisted on the fact that if the breast problem is the result of an act of sorcery, only their intervention will resolve the matter. They recognised instead that "natural" breast problems can also be cured with simpler herbal remedies or with modern medicine.

"If it is through the action of another man that you fall sick, you cannot get better unless a very experienced *guérisseur *treats you. Otherwise, you will treat yourself in vain" (Guérisseur 3, Bambara)

Both women and *guérisseurs *recognised as a first measure to facilitate further treatment, to stroke the breast with the dried primary hand at specific times during the day. In addition, they indicated treating the swelling and the inflammation with a mixture of traditional medical plants such as *Fugufugu *or *Datu *(*Bissap *tree seeds) and *Karité *butter. They explained that a similar healing effect can also be obtained by using river-mud or termites, ants, termites soil, and wasp nests in the preparation of medicaments. Climbing plants are used for medicaments to normalize milk production.

"If the problem is natural, it will be enough to take a bit of termite soil, mix it with potash, and the problem will heal" (22, Marka)

Recognising the complementarities between traditional and modern medicine, the *guérisseurs *wished for a closer collaboration with modern health practitioners.

"Those who say that one is more effective than the other make a mistake, because traditional and modern medicine have same mother and same father" (Guérisseur 3, Bambara)

"We have looked for this collaboration. Some nurses have accepted, but many others have refused" (President of Guérisseurs)

Practices to prevent lactation problems are related to the belief that during pregnancy, in particular among primiparae, two bubbles are formed, one in each breast. If these two bubbles do not burst after delivery, breastfeeding will be difficult and breast problems will develop. Women and *guérisseurs *described both the custom of wearing a tight cloth around the breast and a practice, known as *ecrasement *or *rungri*, which consists of massaging and pulling the breast during pregnancy. To ensure proper lactation, this painful practice is intensified in the weeks following delivery, when the breast is further kneaded with hot water and Karité butter. Women and *guérisseurs *added that other traditional medicaments, primarily mixtures of local plants and Karité butter or baths with potassium, are also regularly applied during pregnancy to prevent breast problems. Furthermore, a few women reported wearing amulets close to their skin.

"There is a bubble in each of the two breasts. If these bubbles do not break, this causes the illness ... To break these bubbles, young mothers must carry a cloth tight around their breasts. Unfortunately, mothers do not like to wear this cloth anymore and so they have problems" (Guérisseur de Goni)

"If this (*ecrasement*) is not done, the bubbles inside the breast will prevent the flow of the milk and this will provoke breast illness" (28, Marka)

In spite of their efforts to care for the breast during pregnancy and following delivery to avoid lactating and health problems, the respondents also acknowledged their incapacity to truly prevent illness. Most respondents in fact, referred to the fact that ultimately God alone can decide over a person's health.

"God alone protects me. Alone, I can do nothing to prevent illness" (21, Fulani).

## Discussion

This study provides an overview of local illness concepts and current health care seeking behaviour in relation to health problems associated with breastfeeding in rural Burkina Faso. Our aim has been that of informing the design of mastitis control measures. Controlling mastitis is in fact desirable both in its own right and in the light of its dangerous potential to increase HIV transmission among breastfed infants [8-13,28].

The first element to deserve attention is that women recognise health problems associated with breastfeeding to be highly prevalent and to constitute a threat to the well-being of both mothers and infants. In addition, although women use a variety of local expressions to define the illness, their recognition of the pathological states associated with a health problem related to breastfeeding is very much in line with biomedical definitions. They identify the same set of states ranging from breast engorgement due to reduced milk flow (statis) to swelling of the mammary glands accompanied by an inflammation (mastitis) and at times developing into a painful breast abscess (infection) recognised by biomedicine. Like women elsewhere in the world, women in the Nouna Health District just do not recognise the existence of sub-clinical mastitis as this, as explained extensively in the introduction, has been defined only in terms of changes, e.g. in breast milk leukocyte numbers and/or in the concentration of pro-inflammatory cytokines, detected through laboratory analysis in the absence of any subjective recognition or symptoms of disease [13,15-17].

In the light of these findings, the dearth of information on the prevalence of mastitis in SSA appears to be almost paradoxical and points at the existence of a large gap between what communities perceive as an important health problem and what clinical and public health specialists have identified as one. Only a handful of studies have attempted to measure the prevalence of mastitis in SSA, generating estimates that range from 10 to 30% [11,15,16]. In order to plan adequate public health interventions to address the problem both within and beyond the framework of PMTCT programs, precise estimates of prevalence based on larger population samples are urgently needed.

Furthermore, the fact that the disease is widely recognised and perceived to constitute an important threat to health indicates that there is a perceived need for treatment and that such need could be translated into actual demand should the necessary conditions, i.e. the provision of culturally sensitive low-cost easily-accessible health services, come into place [33-35]. Likewise, the overlap between the local identification of symptoms and the biomedical definition of mastitis offers health workers an initial "common ground" against which to set educational campaigns and public health efforts aimed at controlling the disease.

What may constitute a challenge to the effective implementation of programs aimed at controlling mastitis is the fact that women, supported by the opinion of local *guérisseurs*, frequently define their breast problems as the product of sorcery or of a supernatural source. Given that one's health care seeking behaviour is inevitably shaped by one's understanding of a disease [36], it does not appear surprising that once women perceive their illness to be "unnatural", they prefer to be treated by a healer. Our findings to this regard are in line with previous findings from SSA, concerning both specific problems related to the breast [29] and women's health care seeking behaviour more in general [37-39].

Bridging a link between biomedical services and traditional healers may serve as an instrument to reach women irrespective of their recognised aetiology of disease. Bridging the link between biomedical services and healers would in fact allow health providers to intervene at the point where traditional medicine is no longer sufficient to treat the disease and still to do so in the respect of local beliefs and practices. The fact that traditional healers also recognise a set of pathological states resembling those defined as relevant by biomedicine coupled with their explicit wish to collaborate with health professionals represent encouraging elements for the set up of a closer collaboration between the two health sectors. Collaborations between healers and biomedical professionals have already been widely implemented in the field of HIV prevention and care [39-43], and there is no reason to believe that they should not be successful also to control mastitis.

The collaboration between traditional healers and biomedical professionals is also essential to secure a community-based intervention to control mastitis. Given that in SSA many women do not come into contact with modern health facilities during their pregnancy [44,45] and that many more women do not test for HIV even in the presence of a PMTCT program [46,47], community-based rather than hospital-based interventions represent a more adequate means of achieving mastitis control and potentially reducing HIV transmission even among women who are unaware of their HIV status. The need for community-based rather than hospital-based interventions is further motivated by the fact that, as reported both by our findings and by prior literature [48], women often do not make their own decisions regarding what care to seek, but act under the influence of older family members and community leaders. Thus, controlling mastitis is only possible if the entire community is involved in the process of prevention and care.

While continued lactation has been observed both in our study area and elsewhere [29,49,50] to be the preferred strategy to outset the development of further breast complications, recent research has identified its dangerous potential to lead to an increase in HIV transmission [8-12]. It has therefore been suggested that HIV positive mothers with breast inflammations should be discouraged from feeding their infants from the affected breast during the period of inflammation [51]. Although recent evidence suggests that EBF may be preferable to substitute feeding even among HIV positive women [21], when suffering from a breast inflammation, HIV positive women should be provided with safe and affordable milk substitutes at least for the time required to treat their condition. Alternatively, due to the fact that the safe and affordable provision of milk substitutes is not always feasible in SSA [21], women could be advised to continue breastfeeding only with the unaffected breast. These strategies, however, have the potential to reach only women who have been identified as HIV positive. Thus, in the light of what stated earlier regarding the possibility that many HIV positive women remain undetected even in contexts where PMTCT programs are available, it is of extreme importance that above all research urgently focuses on developing specific interventions to reduce the incidence of breast inflammations at the community level and subsequently the risk of HIV transmission among breastfed infants.

In particular, specific efforts should be channelled towards assessing the impact of traditional practices, such as the *ecrasement*, and herbal remedies, such as the application of *karité *butter, on the prevention of severe breast problems and on HIV viral load in breast milk. To our knowledge in fact, there is no information available on the effect that traditional practices and African herbal remedies have on mastitis prevention and on viral load in breast milk. If research should show that traditional practices and remedies are effective in draining the breast and lowering HIV concentration in breast milk (thereby reducing the risk of HIV transmission), then such practices and remedies could be promoted within the framework of PMTCT programs. Alternatively, if research should show the opposite effect, educational campaigns would need to actively discourage the adoption of such practices and remedies. This is of great importance given that the traditional practices and remedies reported in our study have also been observed elsewhere [29], suggesting the existence of commonalities in approaches to prevent and treat mastitis across SSA.

## Conclusion

Our study has shown that health problems related to breastfeeding are perceived to be very common in rural Burkina Faso. The spectrum of pathologic states described by the respondents resembles biomedical notions of disease in spite of the fact that the local aetiology differs substantially from the biomedical one. Further studies are needed both to provide an adequate epidemiological picture of mastitis in SSA and to explore the effect of local remedies and practices on the development of severe breast inflammations and on HIV viral load in breast milk. Our study suggests that prevention and care of breast problems related and leading to mastitis should be integrated into PMTCT programs, reaching into the community beyond the boundary of hospital-based interventions and establishing a partnership with traditional healers.

## List of abbreviations

EBF: Exclusive breastfeeding

PMTCT: Prevention of mother to child transmission

SSA: sub-Saharan Africa

WHO: World Health Organisation

## Competing interests

The authors declare no competing interest. The study was supported by the research grant SFB 544 "Control of tropical infectious diseases", Project A6 funded by the German Research Foundation (DFG). The study sponsor had no role in the study design, in the collection, analysis, and interpretation of data, in the writing of the report, and in the decision to submit the paper for publication.

## Authors' contributions

TB and JH were responsible for the conception and design of the study. JH was in charge of the field work, assisted by MS, TB, and MS. MDA and JH analysed and interpreted the data. MDA and MS were in charge of the literature review and drafted the manuscript with contribution from all other authors. All authors read and approved of the final manuscript.

## Pre-publication history

The pre-publication history for this paper can be accessed here:



## Supplementary Material

Additional File 1 Case study: Salima's experience Click here for file
