# Supplementary material for: A qualitative investigation into knowledge, beliefs, and practices surrounding mastitis in sub-Saharan Africa: what implications for vertical transmission of HIV?
Source: BMC Public Health. 2007 Feb 23;7:22. doi: 10.1186/1471-2458-7-22 (PMC1810242; doi:10.1186/1471-2458-7-22)
Supplement: Additional File 1 — Case study: Salima's experience [file 1471-2458-7-22-S1.pdf]

Two weeks postpartum, Salima (a fictional name used to protect the real identity of the women reporting this story), a 29 year-old Marka mother, started to suffer from breastfeeding problems because of breast soreness. Swelling of the right breast quickly got worse and the flow of the maternal milk in the right breast stopped. A few days later, an abscess developed. Salima consulted the hospital to have the abscess removed surgically; there she was also prescribed a course of antibiotics.

Soon afterwards, she had the same symptoms in her left breast, which made Salima no longer able to accomplish her daily tasks as a mother and a housewife. Worried about her own health as well as that of her daughter, she left her husband’s house and moved to her parents’ compound.

To prevent the development of another abscess and to preserve milk flow, her grandmother regularly applied a traditional medicine derived from a plant called *fugu-fugu* onto Salima’s breast. In addition, Salima covered her breast with a fresh preparation of potash several times a day. No other abscess developed, but although Salima continued breastfeeding, her milk was insufficient to feed the infant. The family was convinced that “bubbles” in the breast were obstructing milk flow and called for a *guérisseur*,

The healer asked several questions and examined Salima carefully before reaching a diagnosis. He interpreted a poking, stretching from the armpit along the arm, as a sign of sorcery. He prescribed to Salima a medicine that he himself had produced. It was meant to “neutralize” the power of a negative spirit, which had been sent against the family a few months earlier and which had attacked Salima during pregnancy. Following this “spiritual” treatment, the *guérissuer* also prescribed a physical treatment based on the application of a mixture of herbs and sheabutter.

Salima slowly recovered and resumed normal breastfeeding nine days after treatment.
